# A novel splicing variant in *TECTA* associated with prelingual autosomal dominant nonsyndromic hearing loss via dominant-negative effect

**DOI:** 10.1093/hmg/ddaf109

**Published:** 2025-06-30

**Authors:** Yan Yang, YuanPing Xiong, Hua Lai, Chuanxin Feng, ZhongFa Chen, YaJuan Huang, Zhen Guo, XinYu Li, Laipeng Luo, Feng Zhao, Ping Wu, Haiyan Luo, Yanqiu Liu, Yuhe Liu, Yongyi Zou

**Affiliations:** Jiangxi Key Laboratory of Birth Defect Prevention and Control, Jiangxi Maternal and Child Health Hospital, No. 318, Bayi AvenueDonghu District, Nanchang, Jiangxi Province 330006, China; Department of Otolaryngology Head and Neck Surgery, The First Affiliated Hospital, Jiangxi Medical College, Nanchang University, 17 Yongwaizheng Street, Donghu District, Nanchang, Jiangxi Province 330006, China; Jiangxi Key Laboratory of Birth Defect Prevention and Control, Jiangxi Maternal and Child Health Hospital, No. 318, Bayi AvenueDonghu District, Nanchang, Jiangxi Province 330006, China; Jiangxi Key Laboratory of Birth Defect Prevention and Control, Jiangxi Maternal and Child Health Hospital, No. 318, Bayi AvenueDonghu District, Nanchang, Jiangxi Province 330006, China; Jiangxi Key Laboratory of Birth Defect Prevention and Control, Jiangxi Maternal and Child Health Hospital, No. 318, Bayi AvenueDonghu District, Nanchang, Jiangxi Province 330006, China; Jiangxi Key Laboratory of Birth Defect Prevention and Control, Jiangxi Maternal and Child Health Hospital, No. 318, Bayi AvenueDonghu District, Nanchang, Jiangxi Province 330006, China; Jiangxi Key Laboratory of Birth Defect Prevention and Control, Jiangxi Maternal and Child Health Hospital, No. 318, Bayi AvenueDonghu District, Nanchang, Jiangxi Province 330006, China; Jiangxi Key Laboratory of Birth Defect Prevention and Control, Jiangxi Maternal and Child Health Hospital, No. 318, Bayi AvenueDonghu District, Nanchang, Jiangxi Province 330006, China; Jiangxi Key Laboratory of Birth Defect Prevention and Control, Jiangxi Maternal and Child Health Hospital, No. 318, Bayi AvenueDonghu District, Nanchang, Jiangxi Province 330006, China; Jiangxi Key Laboratory of Birth Defect Prevention and Control, Jiangxi Maternal and Child Health Hospital, No. 318, Bayi AvenueDonghu District, Nanchang, Jiangxi Province 330006, China; Department of Otolaryngology, Head and Neck Surgery, The Second Affiliated Hospital of Nanchang University, 1 Minde Road, Donghu District, Nanchang, Jiangxi Province 330006, People’s Republic of China; Jiangxi Key Laboratory of Birth Defect Prevention and Control, Jiangxi Maternal and Child Health Hospital, No. 318, Bayi AvenueDonghu District, Nanchang, Jiangxi Province 330006, China; Jiangxi Key Laboratory of Birth Defect Prevention and Control, Jiangxi Maternal and Child Health Hospital, No. 318, Bayi AvenueDonghu District, Nanchang, Jiangxi Province 330006, China; Department of Otolaryngology, Head and Neck Surgery, Beijing Friendship Hospital, Capital Medical University, No. 95 Yong’an Road, Xicheng District, Beijing 100050, People’s Republic of China; Jiangxi Key Laboratory of Birth Defect Prevention and Control, Jiangxi Maternal and Child Health Hospital, No. 318, Bayi AvenueDonghu District, Nanchang, Jiangxi Province 330006, China

**Keywords:** hearing loss, TECTA, splicing variant, dominant-negative effect

## Abstract

The *TECTA* gene encodes α-tectorin, the major non-collagenous glycoprotein of the tectorial membrane, and plays a critical role in intracochlear sound transmission. Unsurprisingly, mutations in *TECTA* underlie hearing loss in both mice and humans. Two forms of hearing loss are linked to *TECTA* mutations: DFNA8/12 (autosomal dominant) and DFNB21 (autosomal recessive). Using a combination of clinical examination, pedigree analysis, exome sequencing, and functional studies, we identified a novel aberrant splicing variant, c.5999G > A (p.Gly2000Glu), in *TECTA* as the cause of autosomal dominant hearing loss in five-generation kindred of Chinese descent and provided prenatal diagnosis for the family. To investigate whether the variant acts via a a dominant-negative effect, consistent with pathogenesis observed in mouse models, we performed in vivo RNA analysis. Our data demonstrated that the variant altered RNA splicing, specifically causing aberrant splicing of exon 20 and resulting in two in-frame deletions. Quantitative real-time polymerase chain reaction revealed no significant reduction in mRNA levels in lymphoblasts derived from individuals harboring the *TECTA* c.5999G > A (p.Gly2000Glu) variant or the *TECTA* c.5383 + 6 T > A splicing variant, previously shown to result in exon 16 skipping. This study confirms the involvement of an aberrant splicing mutation in *TECTA* in autosomal dominant nonsyndromic hearing loss, expands the mutational landscape of DFNA8/12 to include coding variants that alter RNA splicing, and underscores the importance of investigating splicing effects of coding variants.

## Introduction

The harmonious interplay between many proteins is required for normal auditory function. Mutations in genes encoding these proteins give rise to hearing loss (HL). The proteins involved in HL often have structurally diverse domains corresponding to distinct functions [1, 2]. Furthermore, some display extraordinary pleiotropy, causing both autosomal-dominant and autosomal-recessive nonsyndromic HL, as is the case with the *TECTA* gene [3, 4]. α-tectorina, the protein product of the TECTA gene, which is non-collagenous glycoprotein that constitutes a major component of the tectorial membrane in the cochlea bridges the stereocilia bundles of specialized sensory hair cells [5, 6]. Sound induces movement of these hair cells relative to the tectorial membrane, deflects the stereocilia, and leads to fluctuations in hair-cell membrane potential, transducing sound into electrical signals. α-tectorin is composed of 3 do-mains: the entactin (ENT) domain, the ZA domain, and the C-terminal zona pellucida (ZP) domain. The ZA domain contains 8 subdomains:1 von Willebrand factor type C domain (vWFC), 4 von Willebrand factor type D domains (vWFD1,vWFD2,vWFD3,and vWFD4), and 3 trypsin inhibitor-like cysteine-richdomains (TIL1, TIL2, and TIL3) [6].

Missense mutations in *TECTA* cause dominant forms of nonsyndromic deafness (DFNA8/12) [7–9], and a genotype–phenotype correlation has been reported in humans, while nonsense mutations cause autosomal-recessive nonsyndromic HL (DFNB21) [10, 11]. The utility of audiogram profiling in heritable HL, to identify candidate gene involvement, has been emphasized by numerous studies [12, 13]. Notably, the phenotype is related to the domain of the *TECTA* mutation.

The extremely high genetic heterogeneity of HL—due to a diverse group of genes encoding proteins essential for the development, function, and maintenance of the complex auditory system—makes genetic diagnosis of this condition challenging [12–14]. Genetic variant classification is crucial for accurate genetic diagnosis [1]. Despite the identification of a large number of variants through next-generation sequencing (NGS), it remains difficult to determine which variants are causative. Therefore, we must consider not only the results of NGS analysis but also the clinical phenotypes of affected individuals. Investigating the effect of coding variants on RNA splicing can provide significant supporting evidence [15–17].

In this study, we identified a sequence variant, c.5999G > A (p.Gly2000Glu), in *TECTA* associated with DFNA8/12 and early-onset autosomal dominant HL in a large family from China. We functionally characterized a novel *TECTA* splicing variant through in vivo RNA analysis and molecular modeling, revealing that dominant-negative effect may be a pathogenic mechanism underlying *TECTA*-associated DFNA8/12.

## Results

### Clinical presentation

**Figure 1:**
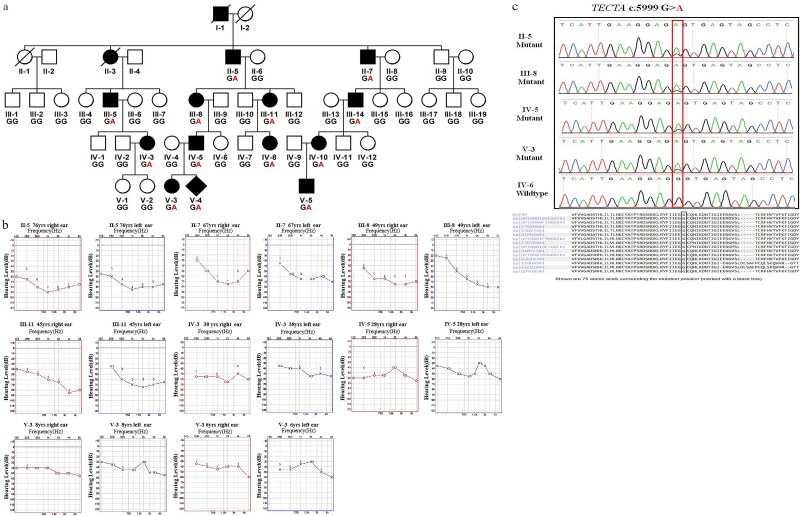
(a) Segregation of the c.5999G > a (p.Gly2000Glu) variant in the family. Circles and squares represent females and males, respectively. Filled symbols denote individuals with non-syndromic HL, and open symbols represent individuals with normal hearing. (b) Audiograms of seven affected individuals at different ages. (c) Representative chromatograms showing wild-type and mutant sequences in *TECTA*.

The proband was IV-5. His wife (IV-4), a healthy pregnant woman, came to the hospital for prenatal genetic counseling because of her husband’s (IV-5) and daughter’s (V-3) HL, which was not associated with any other symptoms. The family pedigree showed five-generation kindred of Chinese descent segregating HL as an autosomal dominant trait (Fig. 1a). There were 14 affected individuals in total. Unfortunately, clinical data could not be collected from I-1 and II-3 because they had died prior to the diagnosis. Seven individuals underwent pure-tone audiometry testing. Individual V-3 was tested twice, at 6 and 8 years of age (Fig. 1b).

In this family, the affected members (II-5, 76 years old; II-7, 67 years old; III-8, 49 years old; and III-11, 45 years old) presented with bilateral severe to profound HL. The HL in II-5 was the most severe, with a bilateral hearing threshold of 84 dB. Individuals IV-3 (38 years old) and IV-5 (28 years old) also reported hearing impairment beginning in their teenage years, but they were not clinically evaluated until adulthood. Individual V-3 passed newborn hearing screening; however, at the age of 6 years, she was diagnosed with HL and had used hearing aids since diagnosis. Audiometry at ages 6 and 8 years revealed stable, moderate, flat-type curve sensorineural HL (Fig. 1b). Phenotypic data could not be collected from the fetus of individual IV-4’s ongoing pregnancy.

Audiograms of the seven affected individuals at different ages showed that HL initially manifested in the high frequencies. Mid-frequency HL was moderate to profound and progressed slowly before reaching a plateau. High-frequency HL remained stable, while mid-frequency HL was characterized by moderate, progressive deterioration. Audiometric profiling in this family revealed a flat configuration before the age of 40 years, shifting to a typical U-shaped pattern in older individuals (Fig. 1b).

Trio whole-exome sequencing (WES), variant filtration, and interpretation.

To identify the genetic etiology of HL in the family, trio WES was performed using DNA samples extracted from the proband (IV-5) and his parents (III-8 and III-9). Single-nucleotide variants, insertions and deletions, and copy number variants were analyzed from the WES-based data. In total, 548 377 single-nucleotide variants and 67 751 insertions and deletions were detected. Additionally, 413 filtered variants were identified in IV-5, 440 in III-8, and 428 in III-9. Variants significantly correlated with the clinical phenotypes were screened, with priority given to identical variants found in the two affected individuals based on the suspected autosomal dominant inheritance pattern observed in the pedigree. Ultimately, three candidate variants were identified: c.5999G > A (p.Gly2000Glu) in *TECTA* (NM_005422.4), c.5471G > A (p.Gly1824Asp) in *TECTA* (NM_005422.4), and c.9950_9951insAT (p.Gly3318Leufs*8) in *ADGRV1* (NM_032119.3) (Table S1). The variant c.5999G > A (p.Gly2000Glu) was present in both affected individuals, IV-5 and III-8.

### Segregation analysis

Segregation analysis was performed in 42 family members to confirm the inheritance pattern of all sequence variants (Supplementary Table S1: Candidate variants found from the WES-based data; Supplementary Table S2: Primer sequences and amplicon sizes for sequence variants). The variant c.5999G > A (p.Gly2000Glu) in *TECTA*, in a heterozygous state, co-segregated with the deafness phenotype in the extended family (Fig. 1a and b).

Splicing analysis.

**Figure 2:**
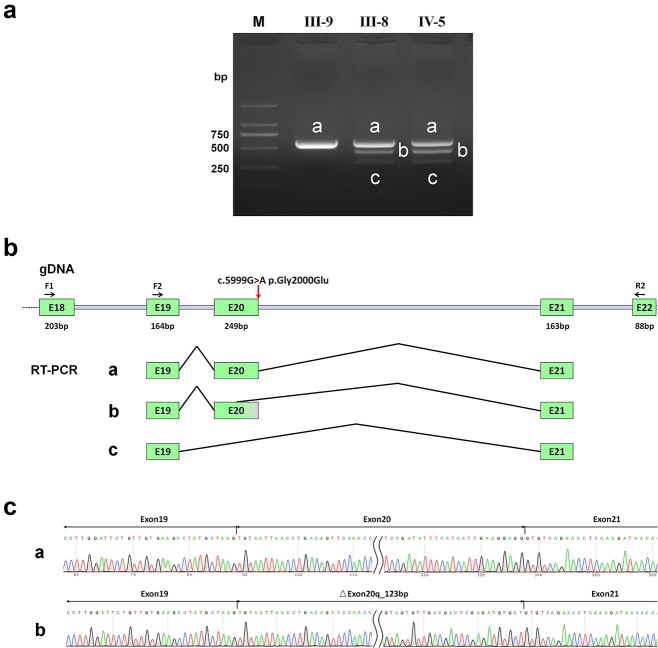
In vivo RNA analysis. (a) Gel electrophoresis of real-time PCR products from a non-affected individual (IV-6) and affected individuals (III-8 and IV-5). M indicates the DNA marker DL2000. The wild-type individual (IV-6) exhibited a single band (band a, 576 bp), while the heterozygous mutant individuals (III-8 and IV-5) showed three bands: Band a (576 bp), band b (453 bp) representing a 123-bp deletion on the right side of exon 20, and band c (327 bp) representing exon 20 skipping. (b) Schematic illustration of aberrant splicing. (c) Sanger sequencing results of the normal and aberrantly spliced transcripts caused by the variant.

**Figure 3:**
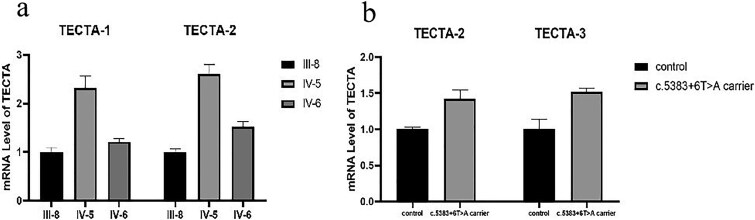
(a) Relative quantification of *TECTA* mRNA levels in lymphoblasts from variant carriers (IV-5 and III-8) and a wild-type individual (IV-6). (b) Relative quantification of *TECTA* mRNA levels in lymphoblasts from an individual carrying the c.5383 + 6 T > a variant and a non-carrier control from the same family. Three biological replicates were included in each group.Three pairs of quantitative primers spanning adjacent exons—TECTA-1, TECTA-2, and TECTA-3—Were used for detection.

The variant c.5999G > A (p.Gly2000Glu) was predicted by SpliceAI to affect the splice site in intron 20, resulting in alteration of the donor site. Our data confirmed that this variant altered RNA splicing and led to two in-frame deletions, as shown by in vivo RNA analysis. Visualization of the splicing products revealed that the wild-type generated the expected 576 bp band containing exon 20 (Fig. 2b, band a). By contrast, the mutant yielded three bands: the expected 576 bp band (band a); a ‘band b’ with an intermediate deletion of 123 bp on the right side of exon 20, leading to a deletion of 41 amino acids; and a ‘band c’ representing complete exon 20 skipping, resulting in a deletion of 83 amino acids (Fig. 2). Sequencing of all bands confirmed the breakpoints and splicing events. Quantitative real-time polymerase chain reaction (PCR) analysis revealed no significant reduction in mRNA levels in lymphoblasts derived from individuals harboring the *TECTA* c.5999G > A (p.Gly2000Glu) variant or the previously reported *TECTA* c.5383 + 6 T > A splicing variant, which results in exon 16 skipping [18] (Fig. 3). Our findings suggest that the pathogenicity of this splicing variant may arise not from nonsense-mediated mRNA decay, but rather from a dominant-negative effect.

**Figure 4:**
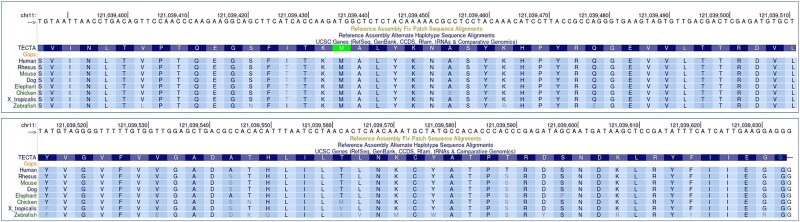
Conservation analysis of *TECTA* c.5999G > a (p.Gly2000Glu) (chr11nn121037374), based on data from the UCSC genome browser (http://genome.ucsc.edu/).

A comparison of the amino acid sequences indicated that the deleted residues caused by aberrant splicing are highly evolutionarily conserved among different species (Fig. 4). According to the latest American College of Medical Genetics and Genomics (ACMG) guidelines [19] and the ClinGen Hearing Loss Specification, we consider this variant to be pathogenic (PVS1 + PM1 + PM2 + PP1_strong + PP4).

### Molecular modeling of the variant in *TECTA*

**Figure 5:**
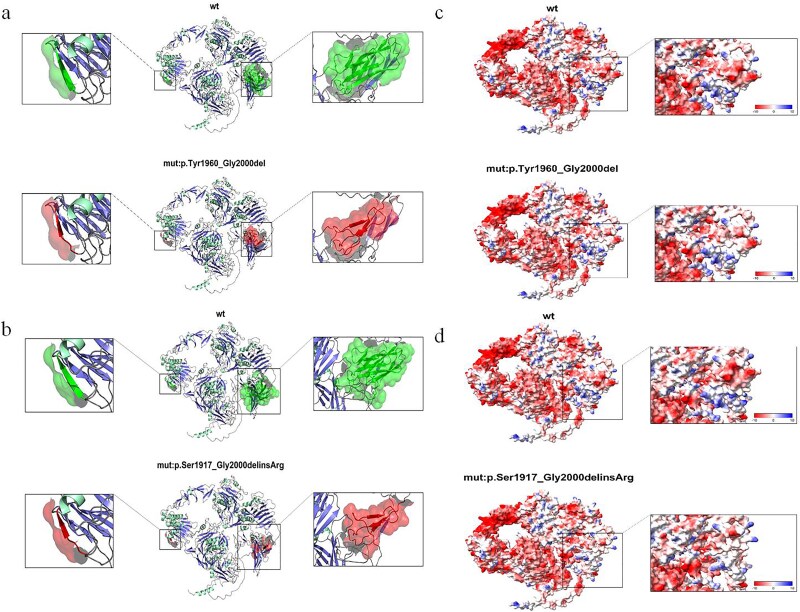
Structural modeling of wild-type and variant TECTA proteins (a, b) Comparative structural models showing wild-type (solid outline) and variant (dashed outline) TECTA proteins. The mutation-affected region is indicated by cross-hatching. (c, d) Three-dimensional surface potential models demonstrating electrostatic changes induced by the variant. Positive potential regions are denoted by plus symbols (+), negative potential by minus symbols (-), and neutral areas remain unmarked.

The structural modeling of the *TECTA* c.5999G > A (p.Gly2000Glu) variant is shown in Fig. 5. This variant created two in-frame deletions and was predicted to result in two abnormal transcripts. In addition to the normal transcript, one abnormal transcript included an intermediate deletion of 123 bp on the right side of exon 20, leading to the loss of 41 amino acids. The wild-type sequence—YVGVFVVGADATHLILTLNKCYATPTRDSNDKLRYFIIE—was deleted in this mutant form. The second abnormal transcript, caused by exon 20 skipping, resulted in deletion of 83 amino acids; the wild-type sequence—SVINLTVPTQEGSFITKMALYKNASYKHPYRQGEVVLTTRDVLYVGVFVVGADATHLILTLNKCYATPTRDSNDKLRYFIIEGG—was removed in the mutant. Using SWISS-MODEL and PyMOL for analysis, we found that the spatial conformation of the protein was altered in the mutant (Fig. 5). Both the 41- and 83-amino acid deletions were located in the C-terminal ZP domain of α-tectorin (Fig. 5a and b). The ZP domain was a 260-amino-acid protein polymerization module found at the C terminus of many secreted eukaryotic glycoproteins that play fundamental roles in development, hearing, immunity, and cancer (InterPro ID: IPR001507). We examined the predicted β-fold structure of *TECTA* and found that the β-fold at the opposite end of the spatial structure was also affected, suggesting that the mutation disrupts the tertiary structure of α-tectorin (Fig. 5a and b). According to analysis using the Adaptive Poisson–Boltzmann Solver in ChimeraX, the protein surface potential was altered by the mutation, with some regions changing from electronegative to neutral. This suggests that the mutation affects the surface potential of α-tectorin (Fig. 5c and d); blue areas indicate positive charge, red areas indicate negative charge, and white areas indicate neutral charge. Protein surface potential is closely related to protein structure and function, and changes caused by the mutation may affect molecular docking where the protein binds to ligands, thereby impacting its normal function [20].

### Prenatal diagnosis

Prenatal molecular diagnosis was performed via amniocentesis at the 18th gestational week. Sanger sequencing revealed that the fetus carried the *TECTA* c.5999G > A (p.Gly2000Glu) variant in a heterozygous state. The decision regarding further action was left to the family.

## Discussion

We employed a combination of WES, cosegregation analysis, in vivo RNA analysis, and molecular modeling to implicate the c.5999G > A (p.Gly2000Glu) variant as the causative factor for moderate to severe mid-frequency and high-frequency sensorineural HL in a large family from China. Our findings suggest that this variant may exert its pathogenic effect through aberrant splicing of exon 20 and subsequent protein misfolding (Fig. 1, Supplementary Table S1).

The variant c.5999G > A (p.Gly2000Glu) in *TECTA*, initially classified as a variant of uncertain significance missense mutation, was first submitted to ClinVar on 26 August 2019 (ClinVar Accession: RCV000487702.2, Variation ID: 666929). Splicing analysis was not conducted at that time because it was not a canonical splicing variant and may not have been supported by sufficient clinical cases. A different base change at the same position, c.5999G > T (p.Gly2000Val), was previously reported as a variant of uncertain significance (PM1 + PM2 + PP3) in a Japanese family [8]. In that case, the proband was a 32-year-old woman with flat, moderate HL, similar to individuals IV-3 (38 years old) and IV-5 (28 years old) in our study. Our data demonstrate that the c.5999G > A (p.Gly2000Glu) variant alters RNA splicing and generates two in-frame deletions, predicted to produce two abnormal transcripts. Molecular modeling revealed that the 83 deleted amino acids are located within the ZP domain of α-tectorin, disrupting the β-fold and altering the protein surface potential in this domain, which may affect protein stability and the binding of interacting partners. To date, *TECTA* gene mutations have mostly been reported in families from Iraq [4], Spain [21], and Japan [22], while the mutation frequency in the Chinese population remains unknown. In this study, we expand the mutation spectrum of *TECTA* and consider this variant to be pathogenic. Our findings also provide evidence (PS1) for pathogenicity assessment based on different variants affecting the same nucleotide position, in accordance with ACMG guidelines.

In humans, mutations in *TECTA* lead to either dominant (DFNA8/A12) or recessive.

(DFNB21) forms of nonsyndromic hearing loss. We reviewed all reported *TECTA* variants associated with hearing loss, compared and contrasted their outcomes, and included the inheritance models and auditory profiles of carriers (Table S3). Missense mutations in *TECTA* that have been reported thus far are associated with the dominant subtype, whereas those leading to recessive deafness are are truncating variants, most likely representing loss-of-function mutations. These distinct mutation patterns between the two inheritance modes correlate with their divergent pathogenic mechanisms. Multiple studies have demonstrated that recessive *TECTA* mutations can lead to nonsense-mediated mRNA decay (NMD). Notably, even truncating variants occurring at the N-terminus of the protein appear to be non-pathogenic [12, 15, 16]. A study of the synonymous variant *TECTA* c.327C > T (p.Gly109=) in seven individuals with HL from six unrelated families showed that in vitro minigene assays disrupted the reading frame of the canonical transcript and introduced a premature termination codon 48 amino acids downstream of the variant, leading to nonsense-mediated decay. These findings suggest that the pathogenic mechanism of *TECTA*-associated autosomal recessive HL is more likely to be loss-of-function [23]. By contrast, *TECTA* knockout homozygous mice—completely lacking α-tectorin—exhibit severe HL characterized by significantly elevated auditory brainstem response thresholds [24]. Anatomical studies of the cochlea in mice harboring a spontaneous *TECTA* missense variant (Ala349Asp/Ala349Asp) revealed complete detachment of the TM from both the organ of Corti and the spiral limbus surfaces [25], accompanied by the absence of striated sheet matrices. Although large quantities of the mutant protein were incorporated into the membrane, they failed to interact with TECTB or otogelin. This missense variant, which primarily causes protein misfolding, resulted in autosomal recessive deafness. Current animal model evidence supports biallelic loss-of-function mutations as the underlying mechanism for recessive *TECTA*-related pathogenesis [26].

Previous studies have speculated that the pathogenic mechanism of ADSNHL associated with *TECTA* mutations is likely dominant-negative [11], though this hypothesis requires further validation through functional assays, animal models, structural biology studies, and expanded clinical data. In the dominant inheritance pattern, pathogenic variants typically consist of missense mutations or splicing variants, Missense variants result in amino acid substitutions, whereas splicing variants produce in-frame deletions that preserve the reading frame. These mutations generate mutant mRNA capable of being translated into aberrant proteins. It has also been reported that the nonsense mutation c.1124delT (p.Val375Alafs*4) can lead to dominant inheritance [21]. In a study by Hildebrand et al. [21], which documented the first known case of homozygosity for a dominant *TECTA* variant, homozygous individuals exhibited severe HL in early childhood in contrast to heterozygous older carriers, who had milder symptoms. Three established dominant genetically engineered mouse models demonstrated that haploinsufficiency is unlikely to explain the heterozygous phenotype [27], as *TECTA* protein was detectable in the TMs of homozygous mutant mice, and heterozygous mice carrying either the *TECTA* DENT allele or complete *TECTA* null alleles exhibited TM morphology nearly identical to wild-type mice. The variant c.1509C > G, which causes autosomal dominant HL, was also investigated in a knock-in mouse model. In this model, the heterozygous TM was attached only to the first row of outer hair cells, while the homozygous TM failed to attach to any outer hair cells.

In our study, skipping of exon 20 in *TECTA* resulted in an in-frame deletion of 83 amino acids in the TECTA protein, leading to protein misfolding and autosomal dominant deafness. Quantitative real-time PCR analysis revealed no significant reduction in mRNA levels in lymphoblasts derived from individuals harboring the *TECTA* c.5999G > A (p.Gly2000Glu) variant or the *TECTA* c.5383 + 6 T > A splicing variant [18]. In a Brazilian family, the *TECTA* c.5383 + 5delGTGA mutation was confirmed to cause exon skipping, leading to autosomal dominant HL. Quantitative real-time PCR analysis in that case revealed only an 11% decrease in *TECTA* mRNA levels in lymphoblasts from the carrier compared with the wildtype [20]. These findings indicate no significant difference in mRNA expression levels between carriers and wild-type individuals. Collectively, these data demonstrate that the in-frame deletion does not trigger nonsense-mediated mRNA decay. We observed elevated *TECTA* mRNA levels in affected individuals (IV-5, Fig. 3), even after accounting for individual variations. This abnormal upregulation may be linked to mutation-induced aberrant splicing. The mutation could activate cryptic splice sites, leading to exon skipping or intron retention, thereby generating truncated yet stable mRNA variants. These variants might escape nonsense-mediated decay (NMD) and produce aberrant α-tectorin proteins within the inner ear tectorial membrane. Such misfolded proteins could disrupt the fibrillar network assembly via a dominant-negative effect, ultimately impairing mechanoelectrical transduction in hair cells and precipitating hearing loss.

Along with observations from human dominant homozygous and dominant animal models, we observed that homozygous individuals exhibited more severe phenotypic manifestations compared to their heterozygous counterparts. This finding and our study supports the hypothesis that a dominant-negative effect, rather than haploinsufficiency, serves as the primary pathogenic mechanism underlying the dominant inheritance pattern of *TECTA* variants.

**Figure 6:**
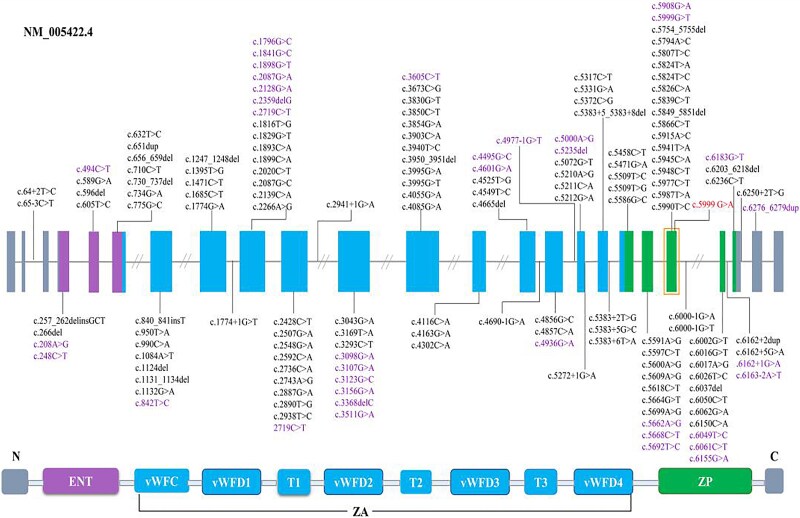
Schematic diagram showing reported pathogenic and likely pathogenic variants in the TECTA gene, alongside the protein structure of α-tectorin. pathogenic variants are indicated in bold font, and likely pathogenic variants are shown in standard font. The c.5999G>A (p.Gly2000Glu) pathogenic variant is specifically highlighted with a solid border and bold formatting. Nucleotide numbering is based on the TECTA transcript NM_005422.4, with the a of the ATG translation initiation site designated as +1. All variants were collected from the deafness variation database (http://deafnessvariationdatabase.org/). Supplementary Table S3 All known TECTA (DFNA8/12 or DFNB21) mutations including those identified in this study.

To date, more than 160 different variants in *TECTA* have been identified in autosomal dominant sensorineural HL, comprising 123 pathogenic and 37 likely pathogenic variants (Fig. 6). Among autosomal dominant forms, DFNA8/12 (*TECTA*) represents the most prevalent subtype in Europe [9], while in Asian populations, genotype–phenotype correlations have been observed with mutations occurring specifically within exons of *TECTA* and its corresponding α-tectorin domains [22]. These mutations are distributed across various domains of the α-tectorin protein, as illustrated in Fig. 6. Specifically, mutations in the N-terminal region—including the entactin domain, von Willebrand factor type D domain 1, and von Willebrand factor type D domain 2—as well as those affecting the ZP domain, are associated with mid-frequency non-syndromic HL. By contrast, missense mutations in the ZA region are commonly linked to high-frequency HL. Previous studies have consistently demonstrated a correlation between the progression of DFNA8/12 HL and specific residue changes, particularly involving cysteine substitutions that may disrupt polypeptide crosslinking [21, 28]. In our study, audiograms of seven affected individuals, ranging in age from 6 to 76 years, revealed an initial manifestation of high-frequency HL, followed by progressive, moderate-to-profound mid-frequency HL that eventually plateaued. These findings align with ARTA analyses of a Belgian-K family carrying a non-cysteine mutation (p.Asn465Lys), which also indicated progressive HL. By contrast, serial audiograms from a Spanish family with the p.Cys1036Tyr mutation in the ZA region exhibited a more stable HL pattern [27]. Our data support previously reported genotype–phenotype correlations in DFNA8/12 and introduce new associations that expand the current understanding of dominant HL penetrance.

Our data highlight the necessity of exercising caution when classifying genetic variants in *TECTA* because different pathogenic mechanisms may be involved. The relationship between domain structure and the inheritance mode of *TECTA* mutations remains inconclusive. Rare missense variants in *TECTA* identified in patients with a DFNA8/12 phenotype should be thoroughly investigated for potential effects on RNA splicing. If functional studies confirm their deleterious impact on splicing, these variants should be reclassified as likely pathogenic. Currently, variant interpretation for coding mutations is primarily focused on predicted effects at the protein level. If variants lie outside canonical splice sites, their potential impact on splicing is often overlooked. However, as demonstrated by several studies [29–32], it is imperative to consider the splicing consequences of coding variants.

In summary, we report and characterize a novel splice-altering variant associated with DFNA8/12, providing evidence that *TECTA* can cause prelingual autosomal dominant nonsyndromic HL via a dominant-negative effect. We expand the mutational spectrum of *TECTA*-related HL to include coding splice-altering variants. Importantly, our data underscore the need for comprehensive variant interpretation, regardless of predicted translational impact, as coding variants may also exert damaging effects on RNA splicing.

A limitation of this study is that the genetic basis of the *TECTA* mutation was explored only through in vivo RNA analysis and molecular modeling; data on protein expression and regulation across tissues are lacking. While our current experimental and functional findings provide substantial evidence, additional independent reports of *TECTA* gene-disease associations would greatly strengthen the validity and generalizability of our conclusions.

## Materials and methods

### Patients and clinical data

A five-generation family of Chinese descent was ascertained as part of a genetic study on dominant progressive HL at Jiangxi Maternal and Child Health Hospital between 2023 and 2024. After obtaining written informed consent from all participants, pure-tone audiograms and relevant medical information were collected from family members. Clinical examination excluded any additional syndromic findings. Blood samples were obtained from 42 individuals, and segregation studies were conducted using Sanger sequencing (Fig. 1a, Supplementary Table S1, Supplementary Table S2). This study was approved by the Ethics Committee of Jiangxi Maternal and Child Health Hospital (EC-KY-2024026).

### Audiological evaluation

No symptoms of vestibular dysfunction were reported. Audiograms were obtained based on each patient’s age and sex. Following otoscopy, rhinoscopy, and oroscopy examinations, pure-tone audiometry was performed to assess air conduction (125–8000 Hz) and bone conduction (250–4000 Hz). Audiograms were available for 7 of the 14 affected individuals in the pedigree (II-5, II-7, III-8, III-11, IV-3, IV-5, V-3) (Fig. 1b).

### WES and bioinformatic analysis pipeline

Exome sequencing was performed on three nuclear family members (III-8, III-9, and IV-5) using the SureSelect Human All Exon V6 kit (Agilent Technologies, Santa Clara, CA, USA), following the manufacturer’s instructions. Paired-end (2 × 150 bp) next-generation sequencing was conducted on the HiSeq 2500 system (Illumina, San Diego, CA, USA) according to the manufacturer’s protocol, achieving an average coverage depth of 138× across 100% of the targeted regions. Raw sequencing reads were filtered using the Trimmomatic program to remove low-quality bases from both ends. Cleaned reads were aligned to the human reference genome (GRCh37) using BWA-MEM. Variant calling was performed using a consensus call method to achieve a balance between high sensitivity and a low false-positive rate. Variant annotation and interpretation were carried out using VEP software, and population frequency data were referenced from the ESP database (Table S1).

### Variant annotations and interpretations

Variant screening was based on the clinical phenotypes of affected individuals, population databases (dbSNP, 1000 Genomes, ExAC), disease databases (OMIM, HGMD, ClinVar), and in silico prediction tools (SIFT, PolyPhen-2, MutationTaster, and SpliceAI). Given the clearly autosomal dominant inheritance pattern of HL in this pedigree (Fig. 1a), raw sequencing data were filtered to retain only rare heterozygous variants associated with autosomal dominant HL in affected individuals. Gene nomenclature follows the conventions of the Human Genome Organization Gene Nomenclature Committee, and variant nomenclature follows the guidelines of the Human Genome Variation Society. Interpretation of variant pathogenicity was based on the most recent guidelines issued by the ACMG and the ClinGen Hearing Loss Specification.

### In vivo RNA analysis

Blood samples from affected and unaffected individuals were collected in BD 762165 PAXgene Blood RNA Tubes to preserve RNA integrity. RNA was isolated using the PAXgene Blood RNA Kit (762174; Qiagen, Hilden, Germany), and cDNA synthesis was performed using random hexamers following standard procedures (GoScript™ Reverse Transcription System, A5000; Promega, Madison, WI, USA). The *TECTA* cDNA segment containing the variant was amplified using either forward primer F1 (5′-TGCAGAGGTGACCTGCAAAG-3′, exon 18) with reverse primer R1 (5′-GTTACACAGCTGCAGAGAGG-3′, exon 23), or forward primer F2 (5′-CCAACAACACTGGCAACATC-3′, exon 19) with reverse primer R2 (5′-AATCCTGGAATTGTGTGGGC-3′, exon 22), using 35 PCR cycles. PCR products were analyzed by agarose gel electrophoresis, excised from the gel, purified, and sequenced as previously described. Quantitative PCR was performed using SYBR Green PCR Master Mix (Applied Biosystems) to assess *TECTA* mRNA levels. The primers used for quantitative PCR were TECTA-1-F (5′-TCTCTGTGGCAACTTCAACG-3′, exon 16) and TECTA-1-R (5′-ATTGTCACACATGGCTGCAT-3′, exon 17); TECTA-2-F (5′-CTGCCACTGAAGCCCTCTAC-3′, exon 9) and TECTA-2-R (5′-GCTTGGCACACAAGAGCATA-3′, exon 9); and TECTA-3-F (5′-GGGGATTACGACGAAGTTCA-3′, exon 21) with TECTA-3-R (5′-CTCACACCCTCCATTGTCCT-3′, exon 23). Two primer pairs, TECTA-2-F/R and TECTA-3-F/R, were used to evaluate *TECTA* mRNA levels in both c.5383 + 6 T > A carriers and controls. All quantitative PCR samples were run in triplicate and normalized to β-actin (*ACTB*) mRNA levels. Each experiment was repeated three times to calculate mean and standard deviation values.

### Molecular modeling

The effects of the mutation on protein conformation were predicted using SWISS-MODEL and PyMOL. Structural models of the mutant protein were typically constructed based on the crystal structure scaffold of the corresponding wild-type protein; alternatively, the three-dimensional structure of a homologous protein was used as a template when necessary [33–35]. To evaluate changes in protein surface potential following the mutation, we performed mapping analysis using the Adaptive Poisson–Boltzmann Solver in ChimeraX.

### Prenatal diagnosis

Prenatal molecular diagnosis was performed through amniocentesis at the 18th gestational week. Fetal amniotic fluid cells were cultured, and DNA was isolated for analysis. The fetal genotype was confirmed by Sanger sequencing.

Statements and Declarations.

## Consent to participate

Informed consent was obtained from all individual participants included in the study.

## Supplementary Material

Table_S1_ddaf109

Tables_S2_ddaf109

Tab_S3_All_known_mutations_of_TECTA-5-9_ddaf109

## Data Availability

The data that support the findings of this study are openly available in *TECTA*c.5999G > A(p.Gly2000Glu) at https://figshare.com/, reference number DOI: 10.6084/m9.figshare.27116728.
