# Supplementary material for: A novel splicing variant in TECTA associated with prelingual autosomal dominant nonsyndromic hearing loss via dominant-negative effect
Source: Hum Mol Genet. 2025 Jun 30;34(18):1517–25. doi: 10.1093/hmg/ddaf109 (PMC12409619; doi:10.1093/hmg/ddaf109)
Supplement: Tab_S3_All_known_mutations_of_TECTA-5-9_ddaf109 [file tab_s3_all_known_mutations_of_tecta-5-9_ddaf109.pdf]

Tab S3 All known TECTA (DFNA8/12 or DF NB21) mutations including those identified in this study

Domain[a]	Mutation[b]	Proteinchang[c]	Frequencies	Inheritance pattern[d]	Progression	Family origin	Reference (PMID)
	c.49_50insT	p.L17Lfs*19)	Unknown	AR	Unknown	Iranian	32864763
	c.208A>G	p.N70D	Mid to High	AD	Stable	Japanese	31554319
	c.257_262delinsGCT	p.Ser86_Pro88delinsCysSer	All freq	AD	Progressive	Chinese	27368438
	c.266delT	p.L89Rfs*34	Mid	AR	Stable	Iranian	21520338
	c.327C>T	p.(G109=)	All freq	AR	Stable	Latin America	35870179
	c.494C> T	p.T165I	Mid	AD	Stable	Japanese	31554319
	c.589G>A	p.D197N	Mid	AD	Stable	American	21520338
	c.596delT	p.L199Rfs*7	Mid	AR	Progressive	Japanese	27368438
NIDO	c.605T>C	p.L202P	Mid	AD	Stable	Japanese	31554319
	c.632T>C	p.F211S	Mid	AD	Stable	Spanish	21520338
	c.651dupC	p.N218Qfs*31	All freq	AR	Stable	Iranian	21520338
	c.654_657delTTTC	p.F219Sfs*12	Unknown	AR	Unknown	UK	27368438
	c.710C>T	p.T237I	High	AD	Stable	Korean	24816743
	c.734G>A	p.W245*	All freq	AR	Unknown	Iranian	28012541
NIDO-vWFC	c.775G>C	p.G259R	All freq	AD	Progressive	Italian	27368438
vWFC-D1	c.950T>A	p.V317E	High	AD	Unknown	Korean	21520338
	c.990C > A	p.Y330*	Unknown	AR	Unknown	Chinese	27368438
	c.1084A>T	p.S362C	Mid	AD	Unknown	American	21520338
	c.1124delT	p.V375Afs*4	Mid	AD	Unknown	Spanish	21520338
vWFD1-	c.1395T>G	p.N465K	Mid	AD	Progressive	Belgian	21520338
TIL1	c.1471C>T	p.R491C	Mid	AR	Progressive	Japanese	27368438
TIL1	c.1685C>T	p.T562M	Mid	AD	Unknown	American	21520338
	c.1816T>G	p.C606G	Mid	AD	Progressive	Japanese	31554319
	c.1893C>A	P.C631*	High	AR	Unknown	Chinese	30703234
	c.1898G>T	p.C633F	Mid	AD	Stable	Japanese	31554319
TIL1	c.2087G>A	p.C696Y	Mid to High	AD	Stable	Japanese	31554319
-vWFD2	c.2087G>C	p.C696S	High	AD	Stable	Japanese	31554319
vWFD2	c.2428C>T	p.R810*	Unknown	AR	Unknown	UK	27368438
	c.2444C>T	p.T815M	Mid	AD	Unknown	American	21520338
	c.2592C>A	p.N864K	Unknown	AR	Unknown	UK	27368438
TIL2	c.2657A>G	p.N886S	High	AD	Progressive	UK	21520338
	c.2941+1G>A	(IVS 9)	All freq	AR	Unknown	Lebanese	21520338
	c.3043G>A	p.E1015K	Mid	AD	Stable	Japanese	31554319
	c.3107G>A	p.C1036Y	Mid	AD	Stable	Spanish	21520338
	c.3123G>C	p.E1041D	Unknown	AR	Unknown	UK	27368438
TIL2-	c.3169T>A	p.C1057S	High	AD	Progressive	Swedish	21520338
vWFD3	c.3293C>T	p.A1098V	High	AD	Unknown	Spanish	21520338

Domain[a]	Mutation[b]	Proteinchang[c]	Frequencies	Inheritance pattern[d]	Progression	Family origin	Reference (PMID)
vWFD3	c.3406G>C	p.D1136H	High	AD	Unknown	Spanish	21520338
	c.3605C>T	p.S1202F	Mid to High	AD	Progressive	Japanese	31554319
	c.3743C>T	p.P1248L	High	AD	Unknown	Spanish	21520338
	c.3743C>T	p.P1248L	High	AD	Unknown	Spanish	21520338
	c.3850C>T	p.R1284C	High / Mid to High	AD	Stable	Japanese	31554319
	c.3903C>A	p.C1301*	Unknown	AR	Stable	Iranian	27368438
vWFD3-TIL3	c.3995G>T	p.C1332F	High	AD	Stable	Korean	25413827
	c.4055G>A	p.C1352Y	Unknown	AR	Unknown	UK	27368438
	c.4495G>C	p.D1499H	Mid	AD	Progressive	Japanese	31554319
vWFD4	c.4525T>G	p.C1509G	High	AD	Progressive	Turkish	21520338
	c.4549T>C	p.C1517R	High	AD	Progressive	Spanish	21520338
	c.4856G>C	p.C1619S	High	AD	Progressive	French	21520338
	c.4857C>A	p.C1619*	Unknown	AR	Unknown	Palestinian	27368438
	c.5072G>T	p.C1691F	Unknown	AR	Stable	Korean	27368438
vWFD4-ZP	c.5210A>G	p.Y1737C	Unknown	AR	Unknown	Iranian	27368438
	c.5211C>A	p.Y1737*	Mid	AR	Stable	Iranian	21520338
	c.5272+1G > A	(IVS15)	All freq	AR	Stable	Algerian	27368438
	c.5317C>T	p.R1773*	Unknown	AR	Unknown	Japanese	27368438
	c.5331G>A	p.R1773*	Mid	AD	Stable	Dutch	21520338
	c.5372C>G	p.P1791R	Mid	AD	Unknown	American	21520338
	c.5383+2T>G	(IVS16)	Mid	AD	Stable	Spanish	21520338
	c.5383+5delGTGA	(IVS16)	High	AD	Progressive	UK	21520338
	**c.5383+6T>A**	**(IVS16)**	**High / Mid to High**	**AD**	**Stable**	**Chinese**	**This sduty 38676628**
	9.6 Kb del		Mid	AR	Stable	Iranian	21520338

Domain[a]	Mutation[b]	Proteinchang[c]	Frequencies	Inheritance pattern[d]	Progression	Family origin	Reference (PMID)
ZP	c.5458C>T	p.L1820F	Mid	AD	Stable	Belgian	21520338
	c.5471G>A	p.G1824D	Mid	AD	Stable	Belgian	21520338
	c.5509T>G	p.C1837G	Mid	AD	Progressive	Spanish	21520338
	c.5509T>C	p.C1837R	Mid	AD	Progressive	American	21520338
	c.5597C>T	p.T1866M	Mid	AD	Progressive / Stable / Unknown	Korean	21520338
	c.5600A>G	p.H1867R	Mid	AD	Progressive	Spanish	21520338
	c.5609A>G	p.Y1870C	Mid	AD	Stable	Australian	21520338
	c.5618C>T	p.T1873I	Mid	AD	Stable	Korean	25413827
	c.5668C>T	p.R1890C	Mid / Mid to High	AD	Stable / Progressive	Dutch / American / Spanish / Korean	21520338<br>30935366<br>31554319
	c.5692T>C	p.C1898R	Mid	AD	Unknown	American	21520338
	c.5807T>C	p.C1898R	Unknown	AD	Stable	Japanese	31554319
	c.5824T>A	p.Y1942N	Mid	AD	Stable	Japanese	31554319
	c.5839C>T	p.R1947C	Mid	AD	Unknown	American	21520338
	c.5945C>A	p.A1982D	All freq	AD	Progressive	Chinese	23936151
	c.5948C>T	p.T1983I	Mid	AD	Stable	Japanese	31554319
	c.5987T>A	p.I1996N	Mid	AD	Stable	Japanese	31554319
	c.5990T>C	p.I1997T	Mid	AD	Progressive	Japanese	27368438
	c.5999G>T	p.G2000V	Mid to High	AD	Progressive	Japanese	31554319
	**c.5999G>A**	p.G2000E	**Mid to High**	**AD**	**Progressive**	**Chinese**	**This sduty**
	c.6016G>T	p.D2006Y	Mid	AD	Unknown	Mongolian	27368438
	c.6026T>C	p.I2009T	High	AD	Stable	Spanish	21520338
	c.6037delG	p.E2013Rfs*6	Mid	AR	Stable	Pakistan	21520338
	c.6062G>A	p.R2021H	Mid	AD	Stable	Japanese	21520338
	c.6162+3insT	(IVS 20)	Unknown	AR	Stable	Korean	27368438
	c.6203_6218del	p.K2068Rfs*38	All freq	AR	Stable	Iranian	21520338
	c.6183G>T	p.R2061S	Mid	AD	Stable	Japanese	31554319

a. NIDO: Nidogen-like domain, ZA: Zonahesin-like domain, VWC: Von Willebrand factor C domain, TIL: Trypsin inhibitor-like domain, VWD: Von Willebrand factor D domain, ZP: Zona pellucida domain,

b. *TECTA* gene sequence [RefSeq: NM_005422.2.4]. Nucleotide numbering reflects cDNA numbering with +1 corresponding to the A of the ATG ranslation initiation codon in the re ference sequence, according to journal guidelines (www.hgvs.org/mutnomen). The initiation codon is codon 1.

c. * nonsense mutation is likely to result in a nonfunctional protein due to premature termination of translation.

d. AD (autosomal dominant) ;AR (autosomal recessive)
